# Treatment non-persistence in children and adolescents with Tourette syndrome newly treated with dopamine D2 receptor modulators

**DOI:** 10.3389/fpsyt.2026.1784601

**Published:** 2026-06-02

**Authors:** Kinga K. Tomczak, Jason P. Swindle, Firas M. Dabbous, Donald L. Gilbert, George B. Karkanias, Sarah D. Atkinson, Frederick E. Munschauer, Faizan Mazhar, Charlotte A. Pettersson, Stephen P. Wanaski, Timothy M. Cunniff, David A. Isaacs

**Affiliations:** 1 Tic Disorders and Tourette Syndrome Program, Department of Neurology, Boston Children’s Hospital, Harvard Medical School, Boston, MA, United States; 2 Thermo Fisher Scientific, Waltham, MA, United States; 3 Division of Neurology, Cincinnati Children’s Hospital Medical Center, Cincinnati, OH, United States; 4 Department of Pediatrics, University of Cincinnati College of Medicine, Cincinnati, OH, United States; 5 Emalex Biosciences, Inc., Chicago, IL, United States; 6 Paragon Biosciences, LLC, Chicago, IL, United States; 7 Department of Neurology, Vanderbilt University Medical Center, Nashville, TN, United States

**Keywords:** antipsychotics, dopamine D2 receptor antagonist, HCRU, side effects, tic disorder, Tourette

## Abstract

**Introduction:**

Dopamine D2 receptor antagonists/partial agonists (D2RAs), including the US Food and Drug Administration–approved Tourette syndrome (TS) agents haloperidol, pimozide, and aripiprazole and other antipsychotics with dopamine D2 receptor–modulating properties, are prescribed for TS. This retrospective real-world study compared adverse events (AEs) and health care resource utilization (HCRU) between pediatric patients with TS newly treated versus not treated with D2RAs.

**Methods:**

Data were analyzed retrospectively from an electronic health records database (TriNetX Dataworks-USA Network) containing information for >119 million individuals. Two cohorts aged 6 to 17 years, from 2011–2021, were identified: a D2RA-exposed cohort indexed at first D2RA medication record with TS diagnosis during baseline (18 months before and including index) and a D2RA-nonexposed cohort indexed on a randomly selected TS diagnosis record (2011–2021) with no D2RA record during baseline or follow-up (18 months after index). Monthly D2RA use was estimated, assuming 30-day coverage into the subsequent month; incident AEs and HCRU were evaluated during follow-up using medication records, diagnosis codes, anthropometric measurements, and/or laboratory results.

**Results:**

Analyses included 1684 individuals per exact-matched cohort. In the D2RA-exposed cohort, documented D2RA medication records declined by Month 3 (38.8% of patients with a D2RA record) through Month 18 (17.9%). After adjustment for measured demographic and baseline characteristics, the D2RA-exposed cohort had higher recorded odds of incident AEs grouped as mild (e.g., sleep disorder; odds ratio [95% CI], 3.36 [2.68–4.20]), moderate (e.g., QT prolongation; 2.30 [1.65–3.20]), and severe (e.g., tardive dyskinesia; 3.01 [2.18–4.16]; all *p* <.0001) compared with the D2RA-nonexposed cohort. The D2RA-exposed cohort also had higher rates of all-cause and TS-related HCRU (outpatient, emergency, and inpatient encounters; adjusted incidence rate ratio range, 1.39–4.10; all *p* <.01).

**Discussion:**

Children and adolescents with TS newly treated with a D2RA showed substantial decline in documented D2RA medication records over follow-up and had higher recorded odds of AEs and HCRU rates than did those without D2RA exposure. These findings should be interpreted as associational rather than causal effects and may reflect residual confounding by indication, differential monitoring, and/or greater underlying disease severity/complexity in the treated cohort.

## 1 Introduction

Tourette syndrome (TS) is a childhood-onset, chronic neurodevelopmental disorder defined by motor and vocal tics that are typically most severe between the ages of 8 to 12 years (1–3). TS has been estimated to affect approximately 0.3% to 0.9% of children and adolescents (1, 4, 5) and approximately 1.4 million individuals in the United States are thought to have TS or another persistent tic disorder (6). Individuals with TS experience impairments in quality of life that span physical, emotional, social, occupational, and cognitive domains and are influenced by both tics and co-occurring psychiatric conditions (e.g., attention-deficit/hyperactivity disorder [ADHD], obsessive-compulsive disorder [OCD]) (7, 8). In addition, risk of death by suicide is increased approximately 4-fold in patients with TS or another persistent tic disorder compared with individuals without either condition (9). Considering the prevalence and burden of TS, safe and effective long-term treatment options are needed.

Aripiprazole, haloperidol, and pimozide are currently approved by the US Food and Drug Administration for the treatment of TS (10). In routine clinical practice, other antipsychotics with dopamine D2 receptor–modulating properties may also be used “off-label” in patients with TS. These dopamine D2 receptor antagonists/partial agonists (D2RAs) are associated with serious adverse effects that can negatively impact quality of life and treatment adherence (2, 10–14). D2RA use is also associated with increased health care resource utilization (HCRU) in both youth and geriatric populations (15–19). Clinical trials of D2RAs in TS have provided useful estimates of adverse events (AEs) such as weight gain (20–22). However, given the wide use of this medication class in youth with TS, real-world estimates of AEs and HCRU in pediatric patients with TS are needed. This study aimed to compare the real-world AE profile and HCRU of children and adolescents with TS who were newly treated versus those who were not treated with D2RAs, as well as to evaluate changes in D2RA treatment patterns over time among those newly treated.

## 2 Materials and methods

### 2.1 Study design and patient identification

This retrospective, observational cohort study was conducted using electronic health records data (2010–2022) from TriNetX Dataworks-USA Network, a database containing information for >119 million unique individuals from 69 US health care organizations (including academic, community-based, integrated, and specialty health care). The patient identification period started on January 1, 2011, and continued through December 31, 2021, with patients identified for inclusion in the study in a sequential manner.

For inclusion in the D2RA-exposed cohort, individuals were required to have ≥1 medication record for D2RA (i.e., aripiprazole, haloperidol, olanzapine, pimozide, quetiapine, risperidone, or ziprasidone) during the patient identification period; were indexed on the date of first D2RA medication record during the patient identification period; and required ≥1 health care encounter with an International Classification of Diseases (ICD), 9th Revision, Clinical Modification (ICD-9-CM) diagnosis code or an ICD, 10th Revision, Clinical Modification (ICD-10-CM) code for TS (ICD-9-CM diagnosis code: 307.23; ICD-10-CM code: F95.2) during the baseline period (18-month period before [and including] the index date). For inclusion in the D2RA-nonexposed cohort, individuals were required to have ≥1 health care encounter with a diagnosis code for TS and were indexed at a randomly selected record with a diagnosis code for TS during the patient identification period. Because a subset of patients in the D2RA-nonexposed cohort had no medication record, a medication-initiation anchor was not available for all members of that cohort; therefore, a randomly selected encounter with a TS diagnosis code was used as a neutral within-care comparison. Patients in both cohorts were required to be 6 to 17 years of age on the index date and to have ≥1 health care encounter with any diagnosis code during both the baseline period and the follow-up period (18-month period after the index date). Patients with ≥1 record for a D2RA medication during baseline were excluded from the D2RA-exposed cohort, and patients with ≥1 record for a D2RA medication during baseline or follow-up periods were excluded from the D2RA-nonexposed cohort, thereby defining a comparator cohort without recorded D2RA exposure during the observed study window.

### 2.2 Standard protocol approvals, registrations, and patient consents

Data were accessed and analyzed using anonymized techniques and were compliant with the Health Insurance Portability and Accountability Act. Approval by an institutional review board or ethics committee and informed consent/assent were not obtained because the retrospective analysis used deidentified data obtained and analyzed in an anonymized fashion, and investigators did not have access to direct patient identifiers. Investigators only had access to the deidentified data elements and analytic outputs available within the TriNetX environment under applicable data-use agreements. No protocol or statistical analysis plan is publicly available for this retrospective database analysis.

### 2.3 Patient characteristics, matching, and outcomes

Patient characteristics included demographics (age, ethnicity, race, geographic region, and sex) at index reported in the source data and baseline body mass index (BMI) category; neuropsychiatric comorbidities (**Supplementary Table 1**) and associated medications (**Supplementary Table 2**); metabolic syndrome severity (**Supplementary Table 3**, **4**); and HCRU (all-cause outpatient, emergency, and inpatient health care encounters; each reported as binary variables) derived from source data. Study variables were defined using diagnosis, medication, laboratory, and anthropometric records available in TriNetX together with the code lists and derivation rules reported in the Methods and Supplementary Tables. These selection and outcome algorithms were chosen for clinical face validity and feasibility within the source data; formal validation studies for the TS-identification algorithm, exposure ascertainment, and several outcome definitions were not conducted. Analyses used the deidentified records as harmonized and available in TriNetX; no additional patient-level chart review or bespoke external data-cleaning step was performed beyond the prespecified cohort definitions, code-based exclusions, and study variable derivations. Patients in each cohort were exact matched with replacement based on age group, index year, geographic region, and sex.

During the follow-up period, incident AEs (i.e., not present during the baseline period, prior to indexing) were identified based on a combination of diagnosis codes, anthropometric measurements (i.e., BMI Z score), and laboratory results. These outcomes were then categorized according to body system (neuropsychiatric, cardiac, metabolic, or hematologic) and severity (**Supplementary Table 5**). This body system/severity framework was developed for this study to organize heterogeneous outcomes clinically and was not intended as a validated adverse-event grading instrument. Neuropsychiatric outcomes, such as OCD, depression, and suicidality, were retained because they are clinically important events that may emerge, worsen, or first be recorded after treatment initiation in routine care; notably, initial documentation of such outcomes in the wake of treatment initiation may reflect underlying TS-related psychiatric comorbidities, evolving disease complexity, and/or increased clinical recognition rather than treatment-emergent drug effects. Levels of AE severity were categorized as follows: *Neuropsychiatric.* Mild: Sleep disorders and OCD. Moderate: Dystonia, akathisia, and other extrapyramidal symptoms (restlessness/urge to move/fidgety/rocking, rigidity/stiffness, bradykinesia, tremor, or postural instability). Severe: Tardive dyskinesia, suicidality (suicidal ideation, suicidal behavior, or suicide attempts), and depression. *Cardiac.* Moderate: Orthostatic hypotension, QT prolongation, and bradycardia/tachycardia. Severe: Malignant arrhythmias and myocarditis/cardiomyopathy. *Metabolic.* Mild: Presence of 1–2 clinical features suggestive of metabolic syndrome (mild metabolic syndrome). Moderate: Presence of 3–5 features suggestive of metabolic syndrome (moderate metabolic syndrome). These clinical features included BMI Z score indicating obesity; hypertension; triglycerides ≥100 mg/dL; high-density lipoprotein cholesterol ≤50 mg/dL; and prediabetes, diabetes, fasting glucose ≥110 mg/dL, or hemoglobin A_1c_ (HbA_1c_) ≥5.7% (**Supplementary Table 3**). Clinical features needed to be present at some point during the follow-up period, even if distributed across multiple health care encounters. *Hematologic.* Moderate: Leukopenia and neutropenia.

Monthly D2RA use was estimated during the follow-up period based on the presence of a D2RA medication record (e.g., prescription or medication order) in a given calendar month, with the assumption that prescriptions provided 30-day coverage extending into the subsequent month. Accordingly, 100% prevalence in Months 1 and 2 was expected by the operational definition. These medication records do not establish actual dispensing or ingestion and may be affected by incomplete capture, out-of-network prescribing, treatment switching, and documentation differences. Incidence rates (per 100 patient-years; allowing multiple events per patient) and incidence rate ratios (IRRs) for all-cause and TS-related HCRU (outpatient, emergency, or inpatient health care encounters) were calculated during the follow-up period. TS-related HCRU was defined as health care encounters with a diagnosis code for TS (ICD-9-CM diagnosis: 307.23 or ICD-10-CM: F95.2).

### 2.4 Statistical analysis

Between-cohort balances in patient characteristics before and after matching were evaluated using standardized mean differences (SMDs; SMD ≥|0.10| indicated meaningful imbalance (23)). The analytic sample comprised all eligible records meeting the prespecified criteria during the database window; no *a priori* sample-size calculation was performed for this retrospective database study. Multivariable logistic regression models evaluated the associations between patient characteristics and incident AEs during the follow-up period, with results reported as odds ratios (ORs) with 95% CIs. Multivariable negative binomial regression models evaluated the associations between patient characteristics and HCRU during the follow-up period, with results reported as IRRs with 95% CIs. Adjustment variables used for regression analyses included baseline age group, ethnicity, race, sex, BMI category, neuropsychiatric comorbidities (**Supplementary Table 1**), medications (**Supplementary Table 2**), severity of metabolic syndrome (**Supplementary Table 3**), and inpatient stay (binary variable). Baseline outpatient and emergency utilization were described but were not included as covariates in the HCRU models. Missingness was handled descriptively as recorded in the source data; no additional imputation was performed. Because meaningful baseline imbalance remained after matching and not all aspects of clinical severity or health care contact could be captured, regression estimates were interpreted as adjusted associational rather than causal effects. Statistical analyses were performed using SAS software, version 9.4 (SAS Institute, Inc., Cary, NC).

### 2.5 Data availability

No protocol, statistical analysis plan, or study-specific programming code is publicly available for this study. Data for this study come from TriNetX Dataworks-USA Network. Data-use agreements with TriNetX stipulate that data may not be shared. Researchers can contact TriNetX to request access to the data used in this study.

## 3 Results

### 3.1 Patient identification and characteristics

**Figure 1:**
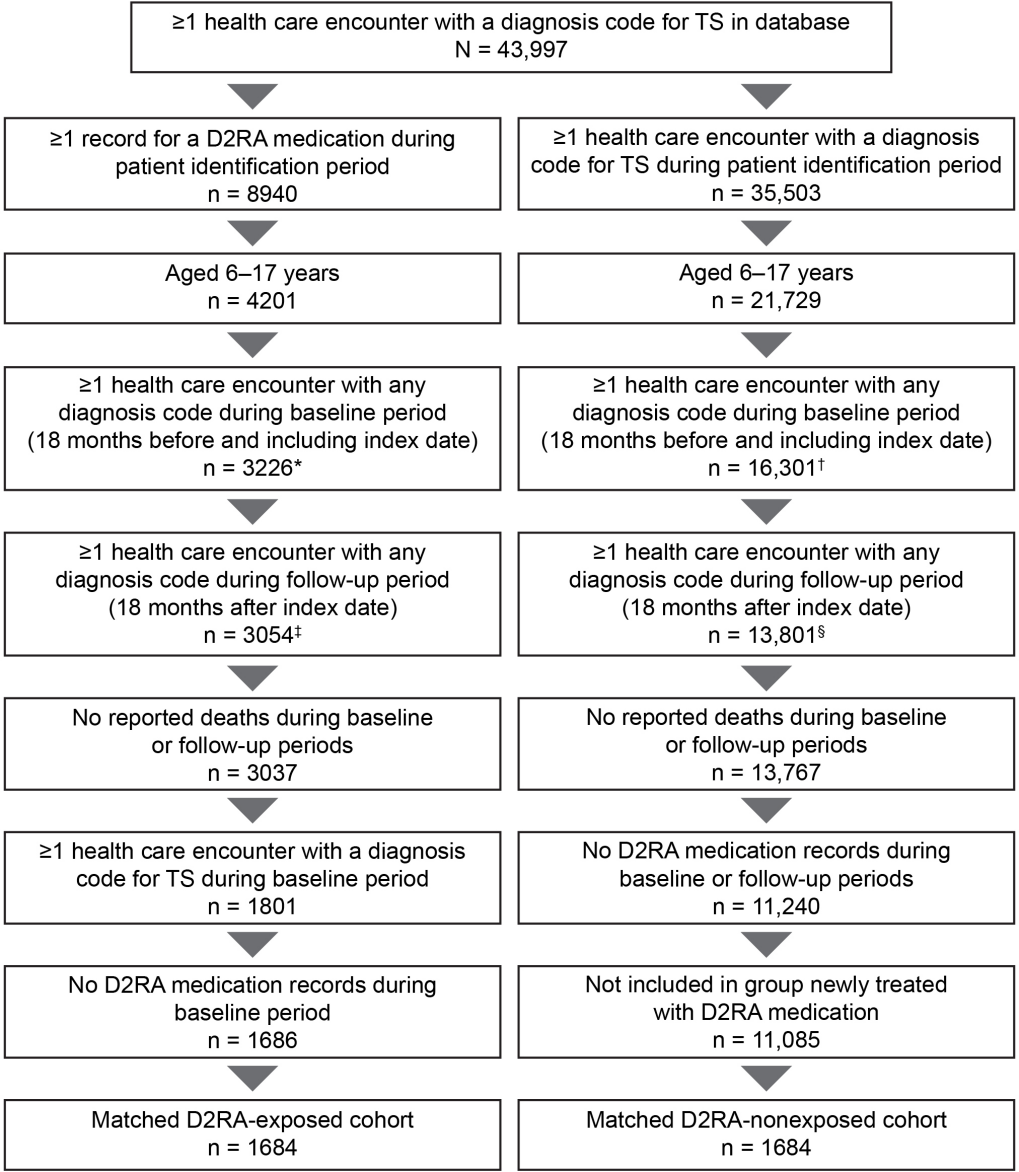
Patient identification. D2RA, dopamine D2 receptor antagonist/partial agonist; TS, Tourette syndrome. *12.3% with unknown/unavailable diagnosis codes. ^†^8.0% with unknown/unavailable diagnosis codes. ^‡^4.1% with unknown/unavailable diagnosis codes. ^§^7.8% with unknown/unavailable diagnosis codes.

A total of 12,771 patients (1686 in the D2RA-exposed cohort and 11,085 in the D2RA-nonexposed cohort) met selection criteria (**Figure 1**). Prior to matching, higher percentages of patients in the D2RA-exposed cohort were aged 12 to 17 years at index and had mild metabolic syndrome, anxiety, ADHD, OCD, depression/mood disorders, autism spectrum disorder, and/or suicidality/suicide attempt during baseline compared with patients in the D2RA-nonexposed cohort (SMD ≥0.17 for all; **Supplementary Table 6**). In addition, higher percentages of patients in the D2RA-exposed cohort had documented use of an alpha-2 adrenergic receptor agonist (clonidine or guanfacine), topiramate, an antidepressant, a stimulant/nonstimulant for ADHD, an antianxiety medication (benzodiazepines and buspirone), and/or an antiseizure medication during the baseline period compared with patients in the D2RA-nonexposed cohort (see **Supplementary Table 2** for categorization of medications; SMD >0.13 for all). Finally, higher percentages of patients in the D2RA-exposed cohort had all-cause outpatient, emergency, and/or inpatient health care encounters during baseline compared with patients in the D2RA-nonexposed cohort (SMD >0.11 for all).

A total of 1684 patients in each cohort were retained for subsequent analyses after matching for age group, index year, geographic region, and sex. These matched cohorts were predominantly male (73.9%) and aged 12 to 17 years (65.8%; **Supplementary Table 6**). Higher percentages of patients in the D2RA-exposed cohort had baseline mild metabolic syndrome, anxiety, ADHD, OCD, depression/mood disorder, autism spectrum disorder, and/or suicidality/suicide attempt compared with the matched D2RA-nonexposed cohort (SMD ≥0.17). In addition, higher percentages of patients in the D2RA-exposed cohort had documented treatment with an alpha-2 adrenergic receptor agonist, topiramate, an antidepressant, a stimulant/nonstimulant for ADHD, an antianxiety medication, and/or an antiseizure medication during the baseline period compared with patients in the matched D2RA-nonexposed cohort (SMD ≥0.17). Finally, higher percentages of patients in the D2RA-exposed cohort had all-cause outpatient, emergency, and/or inpatient health care encounters during baseline compared with the D2RA-nonexposed cohort (SMD ≥0.12).

### 3.2 Monthly D2RA use in the D2RA-exposed cohort

**Figure 2:**
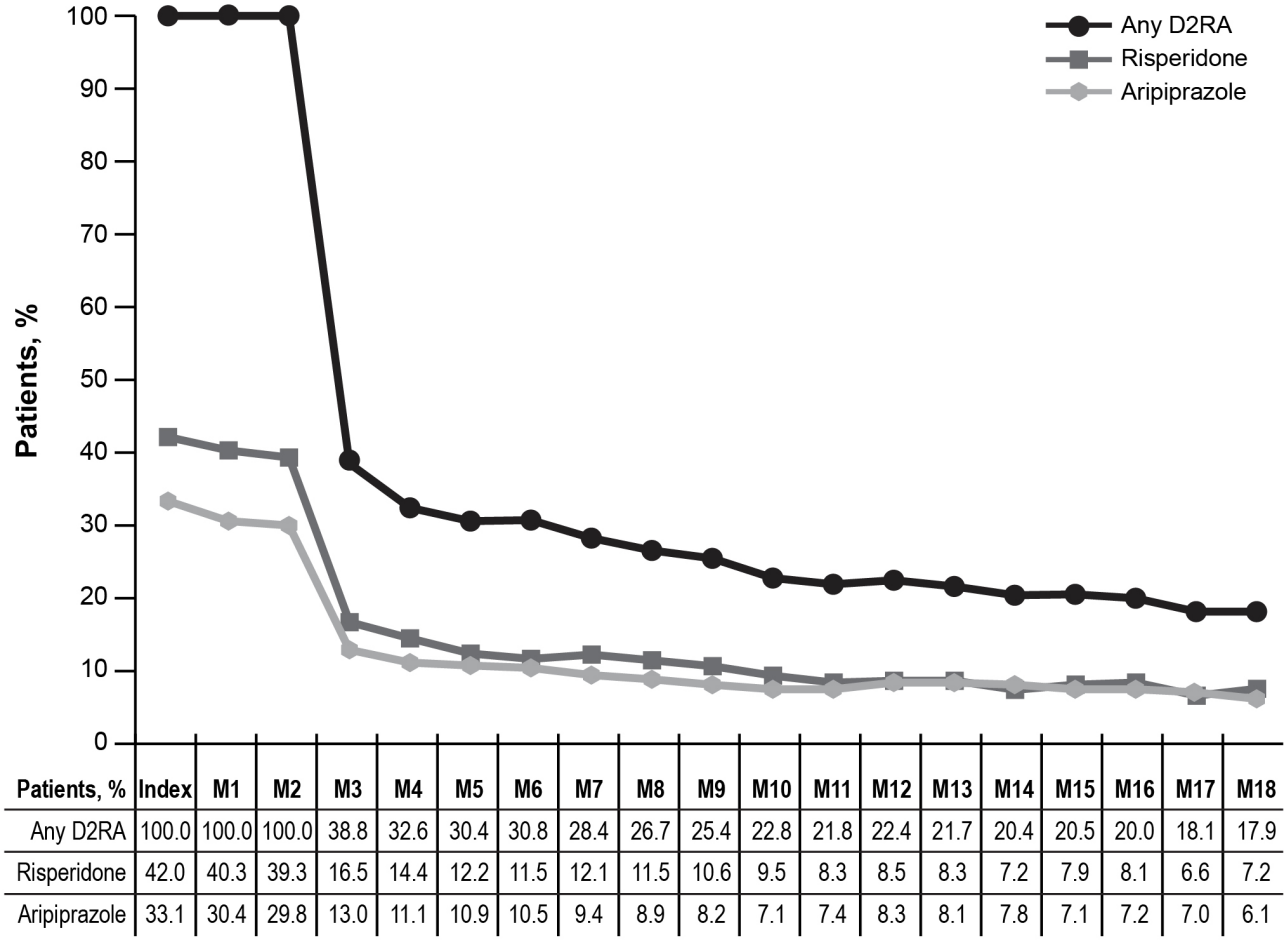
Percentage of patients with medication records for D2RA during the follow-up period.* D2RA, dopamine D2 receptor antagonist/partial agonist; M, month. *D2RA-exposed cohort (*n* = 1684). Evidence of D2RA treatment was based on the percentage of patients with a D2RA medication record in a given calendar month, with the assumption that prescriptions provided 30-day coverage extending into the subsequent month.

On the index date, the most commonly used D2RAs were risperidone (42.0%) and aripiprazole (33.1%) (**Figure 2**). Prevalence rates of D2RA medication records after index were as follows: Months 1 and 2: Any D2RA, 100% and 100% of patients, respectively; risperidone, 40.3% and 39.3%; and aripiprazole, 30.4% and 29.8%. Month 3: Any D2RA, 38.8%; risperidone, 16.5%; and aripiprazole, 13.0%. Month 6: Any D2RA, 30.8%; risperidone, 11.5%; and aripiprazole, 10.5%. Month 12: Any D2RA, 22.4%; risperidone, 8.5%; and aripiprazole, 8.3%. Month 18: Any D2RA, 17.9%; risperidone, 7.2%; and aripiprazole, 6.1%.

### 3.3 Incident adverse events

Incident AEs during follow-up were more commonly recorded in the D2RA-exposed cohort than in the D2RA-nonexposed cohort (**Table 1**). Differences in AEs categorized as mild were observed for sleep disorders (7.7% vs 2.4%; SMD = 0.24), OCD (6.2% vs 2.6%; SMD = 0.18), and mild metabolic syndrome (16.2% vs 3.9%; SMD = 0.42). Differences in AEs categorized as moderate were observed for moderate metabolic syndrome (3.7% vs 0.6%; SMD = 0.22) and bradycardia/tachycardia (3.0% vs 1.5%; SMD = 0.10). Differences in AEs categorized as severe were observed for depression (6.3% vs 2.7%; SMD = 0.18) and suicidality (4.2% vs 1.1%; SMD = 0.19). After multivariable adjustment for measured demographic and baseline characteristics, patients in the D2RA-exposed cohort had higher odds of experiencing a recorded incident AE than did those in the nonexposed cohort across mild (odds ratio, 3.36; 95% CI, 2.68–4.20), moderate (2.30; 1.65–3.20), and severe (3.01; 2.18–4.16; *p* <.0001 for all) categories.

**Table 1:** Summary of incident AEs.

AE	Patients, n (%)		SMD*
	D2RA-exposed (*n* = 1684)	D2RA-nonexposed (*n* = 1684)	
Neuropsychiatric Sleep disorders Depression Obsessive-compulsive disorder Suicidality^†^ Dystonia Akathisia Tardive dyskinesia Other extrapyramidal symptoms^‡^	129 (7.7) 106 (6.3) 105 (6.2) 71 (4.2) 8 (0.5) 3 (0.2) 1 (0.1) 44 (2.6)	41 (2.4) 45 (2.7) 43 (2.6) 19 (1.1) 0 0 0 23 (1.4)	0.24 0.18 0.18 0.19 0.10 0.06 0.03 0.09
Cardiac Bradycardia/ tachycardia Malignant arrhythmias^§^ Orthostatic hypotension QT prolongation Myocarditis/ cardiomyopathy	51 (3.0) 25 (1.5) 8 (0.5) 6 (0.4) 1 (0.1)	25 (1.5) 13 (0.8) 7 (0.4) 1 (0.1) 1 (0.1)	0.10 0.07 0.01 0.07 0.00
Metabolic Mild metabolic syndrome^¶^ Moderate metabolic syndrome^#^	272 (16.2) 63 (3.7)	66 (3.9) 10 (0.6)	0.42 0.22
Hematologic Leukopenia/ neutropenia	8 (0.5)	2 (0.1)	0.07

AE, adverse event; D2RA, dopamine D2 receptor antagonist/partial agonist; SMD, standardized mean difference.

*SMD ≥|0.10| indicates meaningful imbalance. ^†^Suicidal ideation, suicidal behavior, or suicide attempt. ^‡^Restlessness/urge to move/fidgety/rocking, rigidity/stiffness, bradykinesia, tremor, or postural instability. ^§^Including torsades de pointes. ^¶^1–2 metabolic syndrome conditions. ^#^3–5 metabolic syndrome conditions.

### 3.4 Health care resource utilization

**Figure 3:**
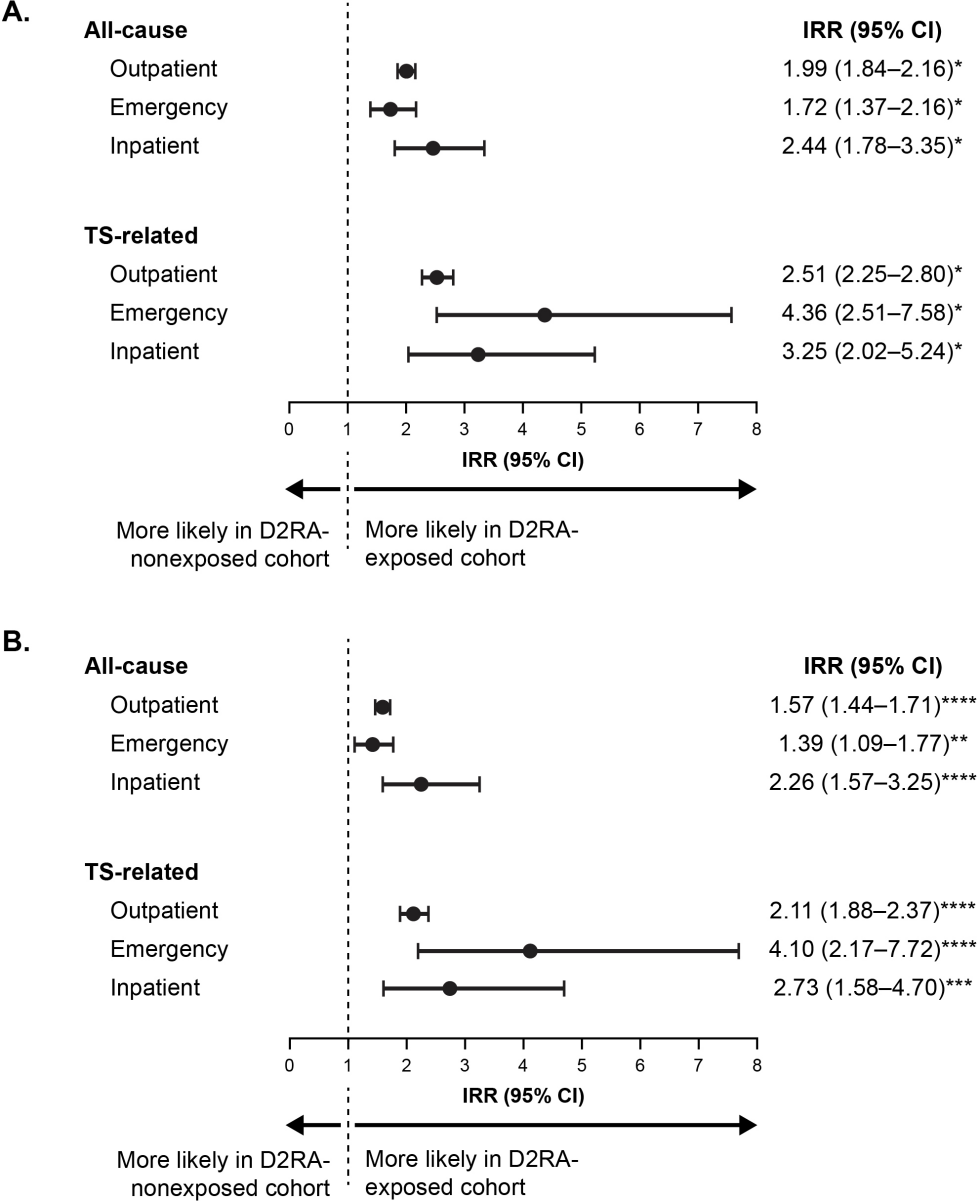
Health care resource utilization during the follow-up period **(A)** before and **(B)** after multivariable adjustment. Health care resource utilization determined with IRRs for all-cause and TS-related (ICD-9-CM diagnosis: 307.23 or ICD-10-CM: F95.2) outpatient (ambulatory, home health, observation, preadmission, or virtual), emergency, and inpatient (hospital, nonacute, or short stay) encounters. D2RA, dopamine D2 receptor antagonist/partial agonist; IRR, incidence rate ratio; TS, Tourette syndrome. *p <.05. **p <.01. ***p <.001. ****p <.0001.

All-cause and TS-related outpatient, emergency, and inpatient health care encounter rates during follow-up were higher in the D2RA-exposed cohort than in the D2RA-nonexposed cohort (IRR range, 1.72 to 4.36; *p* <.05 for all; **Figure 3A**). Multivariable regression analyses yielded similar associations for all-cause and TS-related outpatient, emergency, and inpatient health care encounter rates (IRR range, 1.39 to 4.10; *p* <.01 for all; **Figure 3B**); these results should be interpreted as adjusted associational rather than causal estimates.

## 4 Discussion

In this real-world study, children and adolescents with TS newly treated with a D2RA medication had a rapid decline in documented D2RA medication records after the index date with >60% lacking a subsequent D2RA record by Month 3. The 100% prevalence of D2RA use in Months 1 and 2 was expected from the operational definition because the index medication record and assumed 30-day coverage extended into the subsequent month. Reasons for declining documentation could not be determined from these data and may reflect patient nonadherence, prescriber discontinuation, treatment switching, incomplete capture, out-of-network prescribing, or documentation differences rather than confirmed medication discontinuation. Given the natural history of TS, the expectation in this population is that tic severity or other symptom severity at the index date would still be present 3 months later. Therefore, the magnitude of non-persistence with the only class of medications that are approved for the treatment of TS is striking.

The current study also captured incident AEs and HCRU during an 18-month follow-up period for comparison between the D2RA-exposed and matched D2RA-nonexposed cohorts. This interval was prespecified with the expectation that many patients would continue to have bothersome tics for at least 1 year and would, therefore, continue on D2RA medication. During follow-up, the D2RA-exposed cohort had higher recorded frequencies of incident AEs and higher HCRU than the D2RA-nonexposed cohort, even after adjustment for measured demographic and baseline clinical characteristics. These findings should be interpreted as associational rather than causal. Residual confounding by indication is likely because clinically important baseline differences persisted after matching, and patients selected for D2RA treatment may have had greater tic severity, psychiatric comorbidity, treatment complexity, and/or health care contact than patients in the D2RA-nonexposed cohort. In addition, surveillance or detection bias may have contributed to higher recording of monitored outcomes, such as QT prolongation, metabolic abnormalities, hematologic events, depression/suicidality, and sleep disorders, in the D2RA-exposed cohort. For neuropsychiatric outcomes, such as OCD, depression, and suicidality, first recorded follow-up events may reflect comorbidity recognition or progression of underlying illness rather than treatment-emergent drug effects. Thus, this study identifies a clinically complex, high-need subgroup of youth with TS who were selected for D2RA treatment and had high observed AE and HCRU burdens in routine care.

Percentages of patients with OCD during the baseline period in the current study (25.7% [D2RA-exposed] and 14.4% [matched D2RA-nonexposed]) were lower than a previous estimate for lifetime prevalence of OCD in patients with TS (66.1%) (24). This discrepancy is likely related to patient selection and study design, as the previous study recruited patients from tic disorder specialty clinics and assessed lifetime prevalence of OCD (including subclinical OCD) in clinical interviews (24), whereas the current study assessed prevalence over an 18-month period using ICD codes from electronic health records. Prevalence of ADHD in the current study was consistent with previously published estimates (25, 26).

Higher percentages of patients in the D2RA-exposed cohort had documented treatment with clonidine, guanfacine, and topiramate during baseline compared with the nonexposed cohort. Given that the D2RA-exposed cohort required TS diagnosis before or at index, these results could, in some cases, reflect progression to D2RA treatment in patients who did not respond to alternative TS therapies. However, reasons for medication use could not be determined from this dataset. Initiation of D2RAs should only occur after shared decision-making with patients and their caregivers, consideration of the risks and benefits of these medications, and establishment of a plan to monitor for known neurologic, metabolic, and hormonal adverse effects (2, 27). As noted above, results from the current study highlight the need for alternative long-term therapies for TS. Potential paths forward could include improving access to Comprehensive Behavioral Intervention for Tics, neurostimulation or other nonpharmacologic treatments, and/or drugs with novel mechanisms of action (2). For example, studies of phosphodiesterase inhibitors and cannabinoid-based medications (e.g., SCI-110; NCT05126888) are in phase 2 trials and a phase 3, double-blind, placebo-controlled, randomized withdrawal trial of a dopamine D1 receptor antagonist (ecopipam; NCT05615220) for the treatment of TS was recently completed.

Limitations of the study include its retrospective nature and use of electronic health records data that were not collected specifically to answer the proposed research question, which may have resulted in incomplete capture of medication use, incident AEs, and/or HCRU in the absence of closed claims data. Differential monitoring intensity between cohorts may also have increased ascertainment of laboratory abnormalities, cardiac findings, and psychiatric outcomes in the D2RA-exposed cohort. Individual incident AE intensities and reasons for medication use and D2RA discontinuation could not be determined, and low percentages of patients had laboratory testing results used for categorization of mild or moderate metabolic syndrome, which may have led to an underestimation of incident metabolic AEs. The study also relied on diagnosis-, medication-, laboratory-, and measurement-based algorithms that were not individually validated, which introduced the potential for misclassification. The dataset did not provide standardized assessments, as occurs in clinical trials, did not include additional sensitivity analyses to test alternative confounding-control strategies, and the relative frequencies of identified comorbid conditions should be interpreted with caution (ie, study design may have resulted in over- or under-reporting). In addition, adults with TS were excluded, limiting generalizability to that important group.

In conclusion, children and adolescents with TS newly treated with a D2RA medication had a rapid decline in documented D2RA medication records over follow-up and higher observed AE and HCRU burdens than matched patients without D2RA exposure. These results are descriptive and associational and should not be interpreted as demonstrating causal effects of D2RA treatment. Future research should incorporate richer measures of tic severity and clinical complexity, apply alternative methods for confounding control, and leverage multiple data sources, including integrated claims data and chart reviews, to more clearly differentiate treatment effects from underlying illness burden. Given the prevalence and quality-of-life burden of TS, additional safe interventions with long-term effectiveness are needed.

## Data Availability

The original contributions presented in the study are included in the article/**Supplementary Material**. Further inquiries can be directed to the corresponding author.
