# Supplementary material for: Treatment non-persistence in children and adolescents with Tourette syndrome newly treated with dopamine D2 receptor modulators
Source: Front Psychiatry. 2026 Jun 2;17:1784601. doi: 10.3389/fpsyt.2026.1784601 (PMC13270290; doi:10.3389/fpsyt.2026.1784601)
Supplement: Supplementary file 1 [file Table1.docx]

**Treatment non-persistence in children and adolescents with Tourette syndrome newly treated with dopamine D2 receptor modulators**

Kinga K. Tomczak^1^, Jason P. Swindle^2,^*, Firas M. Dabbous^2^, Donald L. Gilbert^3,4^, George B. Karkanias^5^, Sarah D. Atkinson^5^, Frederick E. Munschauer^5^, Faizan Mazhar^2^, Charlotte A. Pettersson^2^, Stephen P. Wanaski^6^, Timothy M. Cunniff^6^ and David A. Isaacs^7^

^1^Tic Disorders and Tourette Syndrome Program, Department of Neurology, Boston Children's Hospital, Harvard Medical School, Boston, MA, United States; ^2^Thermo Fisher Scientific, Waltham, MA, United States; ^3^Division of Neurology, Cincinnati Children’s Hospital Medical Center, Cincinnati, OH, United States; ^4^Department of Pediatrics, University of Cincinnati College of Medicine, Cincinnati, OH, United States; ^5^Emalex Biosciences, Inc., Chicago, IL, United States; ^6^Paragon Biosciences, LLC, Chicago, IL, United States; ^7^Department of Neurology, Vanderbilt University Medical Center, Nashville, TN, United States

*At the time the study was conducted.

**SUPPLEMENTAL MATERIALS**

**SUPPLEMENTAL TABLE 1.** Diagnosis codes for neuropsychiatric comorbidities

| **Comorbidity** | **ICD-9-CM diagnosis code(s)** | **ICD-10-CM code(s)** |
| --- | --- | --- |
| Abnormal involuntary movements | 781 | R25.9 |
| Anxiety | 309.81, 300.00, 300.01, 300.02, 300.23, 300.09 | F40.1x, F41.0, F41.1, F41.9, F42, F43.1x |
| Attention-deficit/hyperactivity disorder | 314.01 | F90.x |
| Autism spectrum disorder | 299.0x | F84.0x |
| Depression/mood disorder | 311 | F32.x |
| Headache/migraine | 346.XX, 784 | G43.XXX, G43.BX, R51.9 |
| Obsessive-compulsive disorder | 300.3 | F42.x, R46.81 |
| Sleep disorder | 780.5x | G47.x, F51.9 |
| Suicidality/suicide attempt | V62.84, E950-E959 | R45.851, X60-X84, Y87.0 |

ICD-9-CM, International Classification of Diseases, 9th Revision, Clinical Modification; ICD-10-CM, International Classification of Diseases, 10th Revision, Clinical Modification.

**SUPPLEMENTAL TABLE 2.** Neuropsychiatric medications

| **Class** | **Medications** |
| --- | --- |
| Antianxiety | Benzodiazepines,* buspirone |
| Antidepressant | SSRIs, SNRIs, NRIs, NDRIs, NaSSAs, SARIs, SMSs, TCAs, TeCAs, MAOIs |
| Antiseizure^†^ | Acetazolamide, brivaracetam, cannabidiol, carbamazepine, cenobamate, clonazepam, divalproex, felbamate, fenfluramine, lamotrigine, levetiracetam, methsuximide, perampanel, primidone, rufinamide, stiripentol, eslicarbazepine, ethosuximide, everolimus, gabapentin, lacosamide, oxcarbazepine, phenobarbital, phenytoin, pregabalin, tiagabine, vigabatrin |
| Non-D2RA | Botulinum toxin A, clonidine, guanfacine, topiramate |
| Stimulants/nonstimulants for attention-deficit/hyperactivity disorder | Amphetamine, dexamphetamine, methamphetamine, methylphenidate, modafinil, atomoxetine, dexmethylphenidate, lisdexamfetamine, armodafinil, dexmethylphenidate, serdexmethylphenidate |

D2RA, dopamine D2 receptor antagonist/partial agonist; MAOI, monoamine oxidase inhibitor; NaSSA, noradrenergic and specific serotonergic antidepressant; NDRI, norepinephrine-dopamine reuptake inhibitor; NRI, norepinephrine reuptake inhibitor; SARI, serotonin antagonist and reuptake inhibitor; SMS, serotonin modulator and stimulator; SNRI, serotonin-norepinephrine reuptake inhibitor; SSRI, selective serotonin reuptake inhibitor; TCA, tricyclic antidepressant; TeCA, tetracyclic antidepressant.

*Did not include clonazepam, as this was included in the antiseizure medication class. ^†^Did not include topiramate, as this was included in the non-D2RA medication class.

**SUPPLEMENTAL TABLE 3.** Metabolic syndrome conditions

| **Condition** | **Derivation method** |
| --- | --- |
| Abdominal obesity | BMI Z score indicating obesity |
| Hypertension | Diagnosis codes (ICD-9-CM diagnosis: 401.x-405.x; ICD-10-CM: I10-I16, H35.031-H35.033, H35.039) |
| Hyperglyceridemia | Diagnosis codes (ICD-9-CM diagnosis: 272.1; ICD-10-CM: E78.1) or triglycerides ≥100 mg/dL |
| HDL-C ≤50 mg/dL | Per laboratory results |
| LDL-C ≥100 mg/dL | Per laboratory results |
| Prediabetes/diabetes | Diagnosis codes (ICD-9-CM diagnosis: 250.x, 790.29; ICD-10-CM: E08-E13, R73.03), fasting glucose ≥110 mg/dL, or HbA_1c_ ≥5.7% |

BMI, body mass index; HbA_1c_, hemoglobin A_1c_; HDL-C, high-density lipoprotein cholesterol; ICD-9-CM, International Classification of Diseases, 9th Revision, Clinical Modification; ICD-10-CM, International Classification of Diseases, 10th Revision, Clinical Modification; LDL-C, low-density lipoprotein cholesterol.

**SUPPLEMENTAL TABLE 4.** LOINC codes for laboratory test results of interest

| **Parameter** | **LOINC codes** |
| --- | --- |
| Fasting glucose | 101476-0, 10450-5, 104597-0, 104598-8, 14743-9, 14770-2, 14771-0, 1493-6, 1554-5, 1556-0, 1557-8, 1558-6, 17865-7, 35184-1, 41604-0, 41653-7, 59813-6, 76629-5, 77145-1 |
| HbA_1c_ | 17855-8, 17856-6, 41995-2, 4548-4, 4549-2, 55454-3, 59261-8, 62388-4, 71875-9 |
| HDL-C | 12771-2, 12772-0, 14646-4, 18263-4, 2085-9, 2086-7, 27340-9, 35197-3, 49130-8 |
| LDL-C | 12773-8, 13457-7, 18261-8, 18262-6, 2089-1, 2090-9, 22748-8, 35198-1, 39469-2, 49132-4, 55440-2, 69419-0, 96258-9, 96259-7 |
| Total cholesterol | 14647-2, 2093-3, 2565-0, 48620-9 |
| Triglycerides | 12951-0, 14927-8, 1644-4, 17081-1, 2571-8, 3043-7, 3048-6, 3049-4, 30524-3, 35217-9, 47210-0, 70218-3 |

HbA_1c_, hemoglobin A_1c_; HDL-C, high-density lipoprotein cholesterol; LDL-C, low-density lipoprotein cholesterol; LOINC, Logical Observation Identifier Names and Codes.

**SUPPLEMENTAL TABLE 5.** Diagnosis codes for incident AEs

| **System** | **Intensity** | **Incident AE** | **ICD-9-CM diagnosis code(s)** | **ICD-10-CM code(s)** |
| --- | --- | --- | --- | --- |
| Neuro-psychiatric | Mild | Sleep disorders | 780.5x | F51.9, G47.x |
|  |  | Obsessive-compulsive disorder | 300.3 | F42.x, R46.81 |
|  | Moderate | Dystonia | 333.7, 333.6, 333.8 | G24.1, G24 |
|  |  | Akathisia | 333.99 | G25.71 |
|  |  | Other extrapyramidal symptoms* | 333.9, 333 | G26, G25 |
|  | Severe | Tardive dyskinesia | 333.85 | G24.01 |
|  |  | Neuroleptic malignant syndrome | 333.92 | G21.0 |
|  |  | Suicidality^†^ | E950-E959, V62.84 | Y87.0, X60-X84, R45.851 |
|  |  | Depression | 311 | F32.x |
| Cardiac | Moderate | Orthostatic hypotension | 458 | I95.1 |
|  |  | QT prolongation | 426.82 | I45.81 |
|  |  | Bradycardia/tachycardia | 427, 785 | R00.1, R00.0, I47.1, I47.2, I47.9 |
|  | Severe | Malignant arrhythmias^‡^ | 427.xx, 798.x | I49, I47.21 |
|  |  | Myocarditis | 422, 429 | I40, I41, I51.4 |
| Metabolic | Mild | Mild metabolic syndrome^§^ | NA | NA |
|  | Moderate | Moderate metabolic syndrome^¶^ | NA | NA |
| Hematologic | Moderate | Leukopenia | 288 | D70 |
|  |  | Neutropenia | 288 | D70 |

AE, adverse event; ICD-9-CM, International Classification of Diseases, 9th Revision, Clinical Modification; ICD-10-CM, International Classification of Diseases, 10th Revision, Clinical Modification; NA, not applicable.

*Restlessness/urge to move/fidgety/rocking, rigidity/stiffness, bradykinesia, tremor, postural instability. ^†^Suicidal ideation, suicidal behavior, or suicide attempts. ^‡^Including torsades de pointes. ^§^1-2 metabolic syndrome conditions. ^¶^3-5 metabolic syndrome conditions.

**SUPPLEMENTAL TABLE 6.** Patient demographic and baseline characteristics before and after matching

| **Characteristic** | **Before Matching** | | | **After Matching** | | |
| --- | --- | --- | --- | --- | --- | --- |
|  | **D2RA-exposed (n=1686)** | **D2RA-nonexposed (n=11,085)** | **SMD** | **D2RA-exposed (n=1684)** | **D2RA-nonexposed (n=1684)** | **SMD*** |
| Age, mean (SD), y | 12.7 (2.9) | 11.7 (3.0) | 0.37 | 12.7 (2.9) | 12.5 (2.9) | 0.07 |
| Age group, n (%)  6-11 y  12-17 y | 576 (34.2) 1110 (65.8) | 5389 (48.6) 5696 (51.4) | −0.30 0.30 | 576 (34.2) 1108 (65.8) | 576 (34.2) 1108 (65.8) | 0.00 0.00 |
| Sex, n (%)  Male  Female  Missing | 1246 (73.9) 440 (26.1) 0 | 7976 (72.0) 3106 (28.0) 3 (0.03) | 0.04 −0.04 −0.02 | 1245 (73.9) 439 (26.1) 0 | 1245 (73.9) 439 (26.1) 0 | 0.00 0.00 0.00 |
| Race, n (%)  White  Black  Asian  American Indian or Alaska Native  Native Hawaiian or other Pacific Islander  Missing | 1292 (76.6) 87 (5.2) 27 (1.6) 11 (0.7) 4 (0.2) 265 (15.7) | 8116 (73.2) 718 (6.5) 225 (2.0) 50 (0.5) 12 (0.1) 1964 (17.7) | 0.08 −0.06 −0.03 0.03 0.03 −0.05 | 1290 (76.6) 87 (5.2) 27 (1.6) 11 (0.7) 4 (0.2) 265 (15.7) | 1225 (72.7) 105 (6.2) 29 (1.7) 7 (0.4) 4 (0.2) 314 (18.6) | 0.09 −0.05 −0.01 0.03 0.00 −0.08 |
| Ethnicity, n (%)  Not Hispanic or Latino  Hispanic or Latino  Missing | 1428 (84.7) 110 (6.5) 148 (8.8) | 8782 (79.2) 978 (8.8) 1325 (12.0) | 0.14 −0.09 −0.10 | 1428 (84.8) 110 (6.5) 146 (8.7) | 1342 (79.7) 154 (9.1) 188 (11.2) | 0.13 −0.10 −0.08 |
| Geographic region, n (%)  Northeast  Midwest  South  West  Missing | 378 (22.4) 580 (34.4) 537 (31.9) 185 (11.0) 6 (0.4) | 2470 (22.3) 3381 (30.5) 3878 (35.0) 1326 (12.0) 30 (0.3) | 0.00 0.08 −0.07 −0.03 0.02 | 378 (22.4) 580 (34.4) 537 (31.9) 185 (11.0) 4 (0.2) | 378 (22.4) 580 (34.4) 537 (31.9) 185 (11.0) 4 (0.2) | 0.00 0.00 0.00 0.00 0.00 |
| BMI category,^†^ n (%)  Underweight^‡^   Normal weight^§^   Overweight^¶^   Obesity^#^   Missing | 54 (3.2) 653 (38.7) 171 (10.1) 217 (12.9) 591 (35.1) | 332 (3.0) 3859 (34.8) 841 (7.6) 1135 (10.2) 4918 (44.4) | 0.01 0.08 0.09 0.08 −0.19 | 54 (3.2) 652 (38.7) 171 (10.2) 217 (12.9) 590 (35.0) | 55 (3.3) 555 (33.0) 118 (7.0) 168 (10.0) 788 (46.8) | −0.003 0.12 0.11 0.09 −0.24 |
| Metabolic syndrome, n (%)  Unknown  Mild**  Moderate^††^ | 1349 (80.0) 318 (18.9) 19 (1.1) | 9566 (86.3) 1425 (12.9) 94 (0.8) | −0.17 0.17 0.03 | 1347 (80.0) 318 (18.9) 19 (1.1) | 1456 (86.5) 216 (12.8) 12 (0.7) | −0.17 0.17 0.04 |
| Neuropsychiatric comorbidities, n (%)  Anxiety  ADHD  Obsessive-compulsive disorder  Depression/mood disorder  Autism spectrum disorder   Sleep disorder  Headache/migraine  Suicidality/suicide attempt  Abnormal involuntary movements | 999 (59.3) 961 (57.0) 432 (25.6) 276 (16.4) 256 (15.2) 200 (11.9) 148 (8.8) 134 (7.9) 81 (4.8) | 4585 (41.4) 4267 (38.5) 1573 (14.2) 742 (6.7) 938 (8.5) 1025 (9.2) 1137 (10.3) 172 (1.6) 438 (4.0) | 0.36 0.38 0.29 0.31 0.21 0.09 −0.05 0.30 0.04 | 998 (59.3) 960 (57.0) 432 (25.7) 276 (16.4) 256 (15.2) 200 (11.9) 147 (8.7) 134 (8.0) 81 (4.8) | 680 (40.4) 637 (37.8) 242 (14.4) 111 (6.6) 135 (8.0) 137 (8.1) 150 (8.9) 24 (1.4) 82 (4.9) | 0.38 0.39 0.28 0.31 0.23 0.12 −0.01 0.31 0.00 |
| Non-D2RA medications, n (%)  Guanfacine  Clonidine  Topiramate  Botulinum toxin A | 670 (39.7) 423 (25.1) 147 (8.7) 2 (0.1) | 2725 (24.6) 1308 (11.8) 591 (5.3) 6 (0.05) | 0.33 0.35 0.13 0.02 | 669 (39.7) 422 (25.1) 147 (8.7) 2 (0.1) | 386 (22.9) 194 (11.5) 75 (4.5) 2 (0.1) | 0.37 0.36 0.17 0.00 |
| Other medications, n (%)  Antidepressant  ADHD medication  Antianxiety^‡‡^  Antiseizure^§§^ | 923 (54.7) 622 (36.9) 340 (20.2) 223 (13.2) | 2233 (20.1) 2159 (19.5) 821 (7.4) 502 (4.5) | 0.77 0.39 0.38 0.31 | 922 (54.8) 621 (36.9) 340 (20.2) 222 (13.2) | 346 (20.5) 331 (19.7) 122 (7.2) 81 (4.8) | 0.75 0.39 0.38 0.30 |
| Health care resource utilization,^¶¶^ n (%)  Outpatient  Emergency  Inpatient | 1551 (92.0) 294 (17.4) 183 (10.9) | 9829 (88.7) 1201 (10.8) 641 (5.8) | 0.11 0.19 0.18 | 1551 (92.1) 294 (17.5) 181 (10.7) | 1492 (88.6) 185 (11.0) 88 (5.2) | 0.12 0.19 0.20 |

ADHD, attention-deficit/hyperactivity disorder; BMI, body mass index; CDC, US Centers for Disease Control and Prevention; D2RA, dopamine D2 receptor antagonist/partial agonist; SMD, standardized mean difference; TS, Tourette syndrome.

*SMD ≥|0.10| indicates meaningful imbalance. ^†^Based on BMI Z score (calculated per age and sex using CDC growth charts). ^‡^BMI Z score <−1.6. ^§^BMI Z score −1.6 to <1.0. ^¶^BMI Z score 1.0 to <1.6. ^#^BMI Z score ≥1.6. **1-2 metabolic syndrome conditions. ^††^3-5 metabolic syndrome conditions. ^‡‡^Benzodiazepine (did not include clonazepam) or buspirone. ^§§^Did not include topiramate. ^¶¶^Baseline outpatient (ambulatory, home health, observation, preadmission, or virtual), emergency, and inpatient (hospital, nonacute, or short stay) encounters reported as binary variables.
